# Small nucleolar RNA Sf-15 regulates proliferation and apoptosis of *Spodoptera frugiperda* Sf9 cells

**DOI:** 10.1186/s12867-019-0128-9

**Published:** 2019-04-11

**Authors:** Bo Wu, Lei Huang, Wujie Qiu, Xiao Liu, Yawen Shen, Yiping Lu, Zonglin Yang, Xinmei Li, Bin Cui, Shidong Xu, Huili Qiao, Reng Qiu, Lunguang Yao, Yunchao Kan, Dandan Li

**Affiliations:** China-UK-NYNU-RRes Joint Laboratory of Insect Biology, Henan Key Laboratory of Insect Biology in Funiu Mountain, Nanyang Normal University, 1638 Wolong Road, Nanyang, 473061 Henan China

**Keywords:** snoRNA, Sf-15, Proliferation, Transcriptome, Sf9 cells

## Abstract

**Background:**

Small nucleolar RNAs (snoRNAs) function in guiding 2′-*O*-methylation and pseudouridylation of ribosomal RNAs (rRNAs) and small nuclear RNAs (snRNAs). In recent years, more and more snoRNAs have been found to play novel roles in mRNA regulation, such as pre-mRNA splicing or RNA editing. In our previous study, we found a silkworm C/D box snoRNA Bm-15 can interact with *Notch* receptor gene in vitro. To further study the function of Bm-15, we cloned its homolog Sf-15 from *Spodoptera frugiperda* and investigate the function of Sf-15 in Sf9 cells.

**Results:**

We showed that knocking down of Sf-15 can inhibit the proliferation, then induce apoptosis of insect *S*. *frugiperda* Sf9 cells, but the results were reversed when Sf-15 was overexpressed. De novo sequencing of transcriptome of Sf9 cells showed that the expression of 21 apoptosis-related genes were increased upon Sf-15 repression. Further analysis showed that a Ca^2+^-induced cell death pathway gene *Cn* (PPP3C, the serine/threonine-protein phosphatase 2B catalytic subunit), was significantly increased upon Sf-15 depression but decreased when Sf-15 was overexpressed, which indicated that *Cn* might be a potential target of Sf-15.

**Conclusions:**

We conclude that C/D box snoRNA Sf-15 can participate in apoptosis through regulating the expression of Ca^2+^-induced cell death pathway gene *Cn* in Sf9 cells. This is the first time that we found snoRNAs exhibiting dual functions in insect, which reveals a novel layer of ncRNA modulation in cell growth and death.

**Electronic supplementary material:**

The online version of this article (10.1186/s12867-019-0128-9) contains supplementary material, which is available to authorized users.

## Background

Small nucleolar RNAs (snoRNAs) are one of the most abundant non-coding RNA species of 60–300 nucleotides (nt) length and function in guiding 2′-*O*-methylation (mediated by C/D box snoRNAs) and pseudouridylation (by H/ACA box snoRNAs) in ribosomal RNAs (rRNAs), small nuclear RNAs (snRNAs), tRNAs, and mRNAs [1, 2]. However, in recent years, a variety of snoRNAs have been found to display novel functions, such as processing molecules into smaller fragments [3–12] and playing regulatory roles in transcriptional or post-transcriptional levels in human diseases [13–24]. For example, the brain-specific snoRNA, HBII-52, is involved in the regulation of RNA editing and pre-mRNA splicing of serotonin receptor 2C (5-HT_2C_R) through an 18 nt complementarity to the 5-HT_2C_R pre-mRNA, and plays important roles in Prader-Willi syndrome [17–19, 25–28]. Furthermore, accumulating evidence suggests that dysregulation of snoRNAs contribute to tumorigenesis [20, 29–32]. For example, accumulation of gas5-generated snoRNAs was related to growth arrest of breast cancer cells [12, 33]. Inhibition of tumor-abundant SNORD78 suppressed the proliferation of non-small cell lung cancer (NSCLC) cells via inducing G0/G1 cell cycle arrest and apoptosis, while SNORD78 overexpression promoted cell proliferation [15]. Additionally, SNORD113-1 was found to inactivate the phosphorylation of ERK1/2 and SMAD2/3 in MAPK/ERK and TGF-β pathways and suppress tumorigenesis in hepatocellular carcinoma [34], which indicated that the biological function of snoRNAs are various and elusive.

SnoRNAs in lepidopterous insects of silkworm *Bombyx mori* have been studied by Li et al. [2], but their functional roles have not been fully elucidated. In a previous study, we found a C/D box snoRNA, Bm-15, can interact with a *Notch* receptor gene in vitro [2]. To further study the function of this snoRNA, we cloned its homolog from *Spodoptera frugiperda* Sf9 cells, we found that Bm-15 was highly conserved between *B*. *mori* and *S*. *frugiperda*. Further analysis showed that suppression of Sf-15 inhibited cell growth and promoted cell apoptosis. Transcriptome analysis showed that a serine/threonine-protein phosphatase gene *Cn* was a potential target of Sf-15. This is the first report that a lepidopterous snoRNA can participate in cell growth through Ca^2+^-induced cell death pathway, which may provide new clues for understanding the function of snoRNAs.

## Results

### Detect the presence of Bm-15 in Sf9 cell

**Fig. 1:**
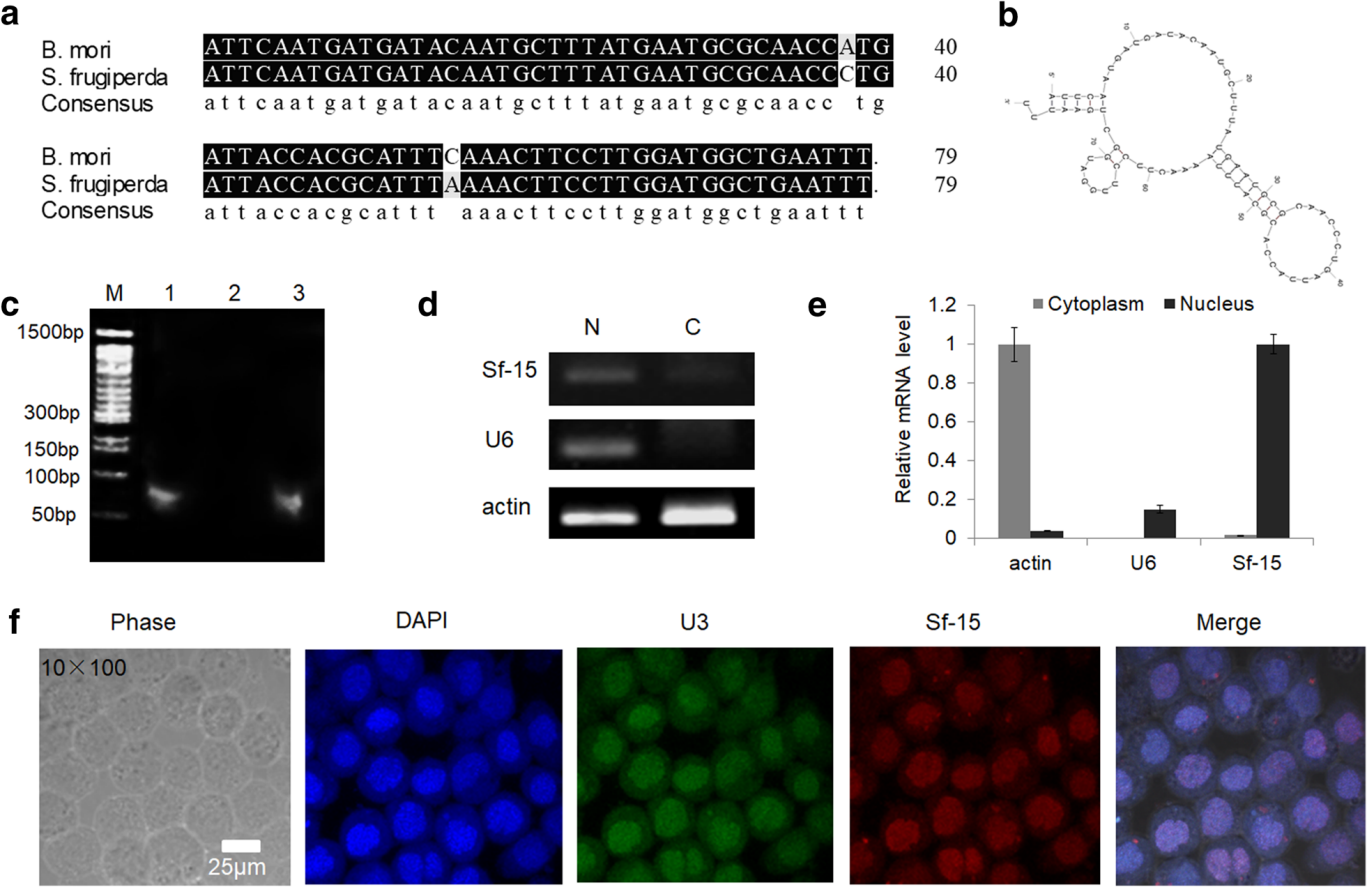
Characterization of Sf-15 in Sf9 cells. **a** Sequence identity of Bm-15 between *B*. *mori* and *S*. *frigiperda*. **b** Secondary structure of Sf-15 in *S*. *frigiperda*. **c** Abundance of Sf-15 that was pulled down by the antibody of Fibrillarin. M, 100 bp DNA ladders. 1–3 represented PCR products that were amplified from RNAs being pulled down by anti-Fibrillarin, serum and input, respectively. **d** Expression of Sf-15 in nucleus and cytoplasm of Sf9 cells. *N* Nucleus, *C* cytoplasm. **e** Relative expression of Sf-15 in nucleus and cytoplasm by quantitative real-time PCR. Results were calculated by relative Ct of different genes. **f** Fluorescent in situ hybridization of Sf-15. Phase represented cells observed under white light. U3 snoRNA was used as a nucleolar marker and was visualized by employing a 5′-FITC-labeled antisense oligonucleotide. The in situ hybridization probes of Sf-15 were labeled with Cy3 at the 5′ end, the nucleus was stained with DAPI. Scale bar represented 25 μm

In our previous studies, we found that a C/D box snoRNA Bm-15 can interact with a *Notch* receptor gene in *B*. *mori*. To further study the function of Bm-15, we cloned its homolog in *S*. *frugiperda* Sf9 cells (whose transfection efficiency is much higher than cell lines of silkworm). We found that Bm-15 was conserved between *B*. *mori* and *S*. *frugiperda*, with sequence identity of 97% and similarly secondary structure (Fig. 1a, b). Eukaryotic C/D box snoRNAs guide 2′-*O*-methylation of rRNAs, snRNAs, and tRNAs through C/D box small nucleolar ribonucleoprotein particles (C/D box snoRNPs), which involves the core proteins of Snu13p/15.5 K, Nop58p/NOP58, Nop56p/NOP56, and Nop1p/Fibrillarin. To determine the characteristics of Sf-15, we used the antibody against Fibrillarin, one of the most important core protein of C/D box snoRNPs to pull down RNAs. Results showed that Sf-15 can be fished out by Fibrillarin (Fig. 1c), which suggested that Sf-15 might be a real snoRNA.

Next, the cellular location of Sf-15 was detected in Sf9 cells. Results showed that Sf-15 was highly existed in the nucleus of Sf9 cells (Fig. 1d, e). Moreover, Immunofluorescence in situ hybridization (FISH) of Sf-15 with Cy3-labeled antisense probes confirmed that Sf-15 was dominantly in nucleus of Sf9 cells (Fig. 1f).

### Repression of Sf-15 blocks proliferation and promotes apoptosis and death of Sf9 cells

**Fig. 2:**
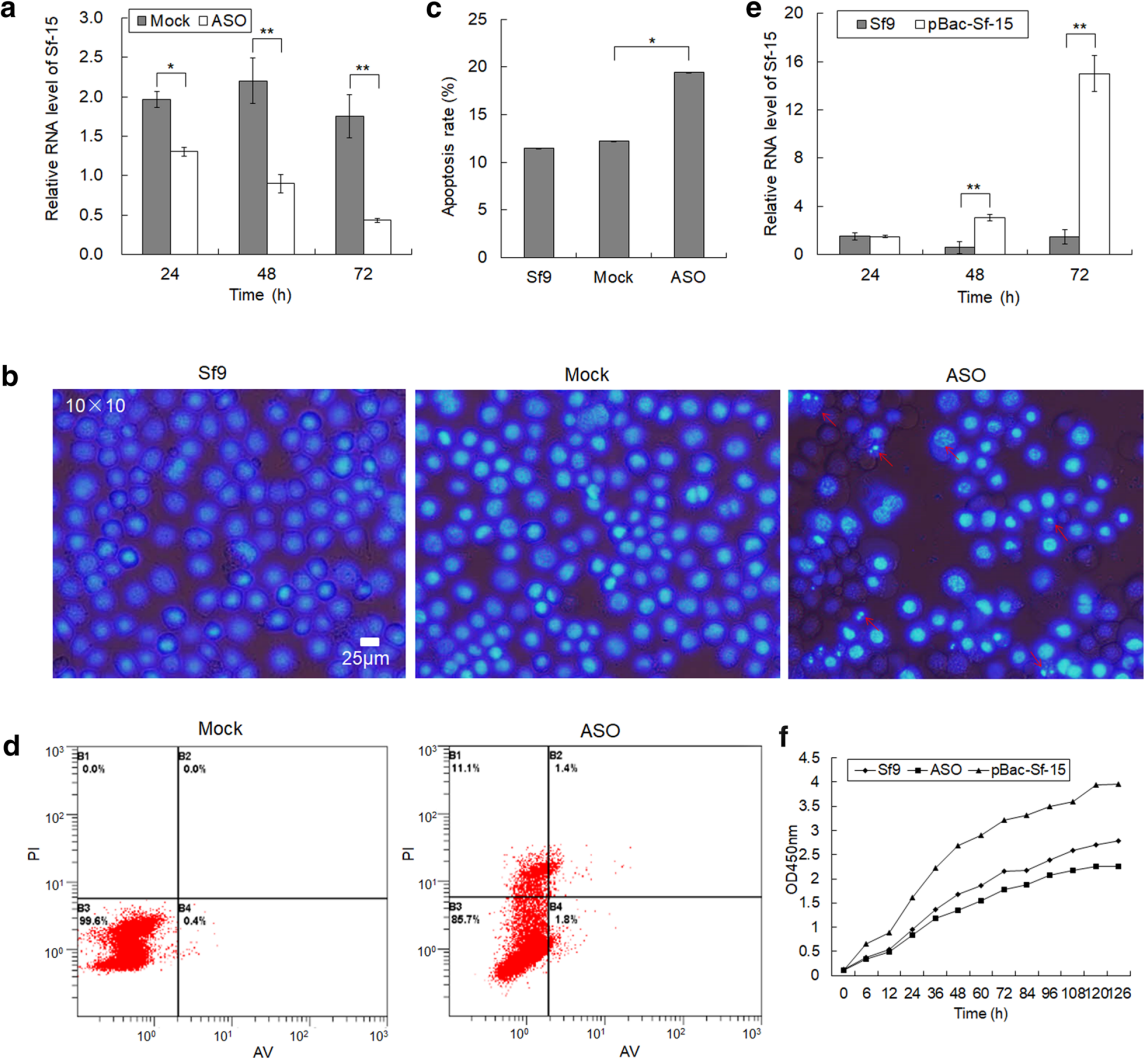
Knocking down of Sf-15 inhibited the proliferation and induced apoptosis and death of Sf9 cells. **a** Relative expression of Sf-15 after ASO transfection at different time-points. Mock means Sf9 cells transfected with antisense oligonucleotide of negative control (NC), ASO menas cells transfected with antisense oligonucleotides of Sf-15. U6 was used as an internal control. **b** DAPI staining showed chromatin condensation and apoptotic bodies in Sf9 cells after 72 h transfection of Sf-15 antisense oligonucleotide. Sf9 represented normal Sf9 cells, Mock and ASO refers to cells transfected with ASO of NC and Sf-15, respectively. Red arrows showed the apoptotic bodies. Scale bar represented 25 μm. **c** Apoptosis rate of cells that were transfected with antisense oligonucleotide of NC (Mock) and Sf-15 (ASO), respectively. **d** Apoptotic rates determined by Annexin-V/PI stain after transfection with antisense oligonucleotide of NC (Mock) and Sf-15 (ASO), respectively. The B3, B2, B4 and B1 quadrants in each panel represent the populations of normal, early and late apoptotic, and apoptotic necrotic cells, respectively. **e** Relative expression of Sf-15 after transfection of *pBac[A3*-*EGFP*-*A3*-*Sf*-*15]* (*pBac*-*Sf*-*15*) vector at different time-points. U6 was used as an internal control. **f** Growth curves of Sf9 cells detected by WST-8 method upon Sf-15 overexpression and repression. 6, 12, 24, 36, 48, 60, 72, 84, 96, 108, 120 and 126 h represented different timepoint after transfection of Sf-15 antisense oligonucleotides (ASO) and *pBac[A3*-*EGFP*-*A3*-*Sf*-*15]* (*pBac*-*Sf*-*15*) respectively. Sf9 means normal Sf9 cells

snoRNAs are located in the nucleoli, the traditional methods of RNA interference (RNAi) such as dsRNAs or siRNAs can hardly work, but RNase H1-dependent antisense oligonucleotides (ASOs) can be easily transported into the nucleus [35–38]. So here the modified ASO of Sf-15 was used to repress the expression of Sf-15 in Sf9 cells. Results showed that the expression of Sf-15 was inhibited by nearly 33% after 24 h transfection, and reached to 75% after 72 h transfection of ASO (Fig. 2a), which indicated that the modified ASO can effectively knock down the expression of Sf-15.

Cell morphology observations with DAPI showed that Sf9 cells with chromatin condensation and apoptotic bodies increased by approximately 9% after 72 h transfection of Sf-15 antisense oligonucleotides (Fig. 2b, c, number of examined cells of each sample is 1500). Moreover, flow cytometry assay revealed that the apoptotic rate of Sf9 cells increased by approximately 3.2%, and the death rate of cells increased 11.1% after 72 h transfection of Sf-15 ASO (Fig. 2d).

To further study the regulator roles of Sf-15 in cell growth, the overexpression vector *pBac[A3*-*EGFP*-*A3*-*Sf*-*15]* was used. Results showed that the expression of Sf-15 increased 5 times after 48 h transfection, while to 10 times after 72 h transfection (Fig. 2e), which indicated that the Sf-15 was successfully overexpressed. Then the cell proliferation rate was detected by WST-8 (the analogue of MTT), results showed that the cell growth rate was impaired by nearly 19% after 48 h transfection of Sf-15 ASO, but increased 38% as ectopic overexpression of Sf-15 at the same time-point (Fig. 2f), which indicated that Sf-15 might participate in the regulation of cell proliferation.

### Transcriptome analysis of knocking down and ectopic expression of Sf-15 in Sf9 cells

To determine the roles that Sf-15 plays in cell growth, RNA libraries of normal Sf9 cells (named Sf9), cells with repression (named ASO) and overexpression of Sf-15 (named Sf-15) were constructed respectively. De novo transcriptome sequencing was used to detect the differential expression of protein coding genes. A total of 59,297,730, 59,614,986 and 57,045,606 raw reads were obtained from the three libraries of Sf9, ASO and Sf-15, respectively. After removing the low-quality reads and adaptor sequences, a total of 77,578, 82,715, and 73,398 contigs were obtained from the three libraries, respectively (Table 1). Finally, we got 46,529, 47,231 and 43,032 unigenes from Sf9, ASO and Sf-15, respectively, with an average length of 750 bp.

**Table 1:** Summary of De novo transcriptome sequencing

Samples	Total raw reads	Total clean reads	Total clean nucleotides (nt)	Contig number	Contig length (nt)	Mean contig length (nt)	Total unigene number	Total unigene length (nt)	Mean unigene length (nt)
Sf9	59,297,730	55,266,566	4,973,990,940	77,578	30,360,233	391	46,529	35,208,893	757
ASO	59,614,986	55,185,956	4,966,736,040	82,715	31,014,680	375	47,231	36,395,105	771
Sf-15	57,045,606	52,924,702	4,763,223,180	73,398	27,797,369	379	43,032	31,685,987	736

**Fig. 3:**
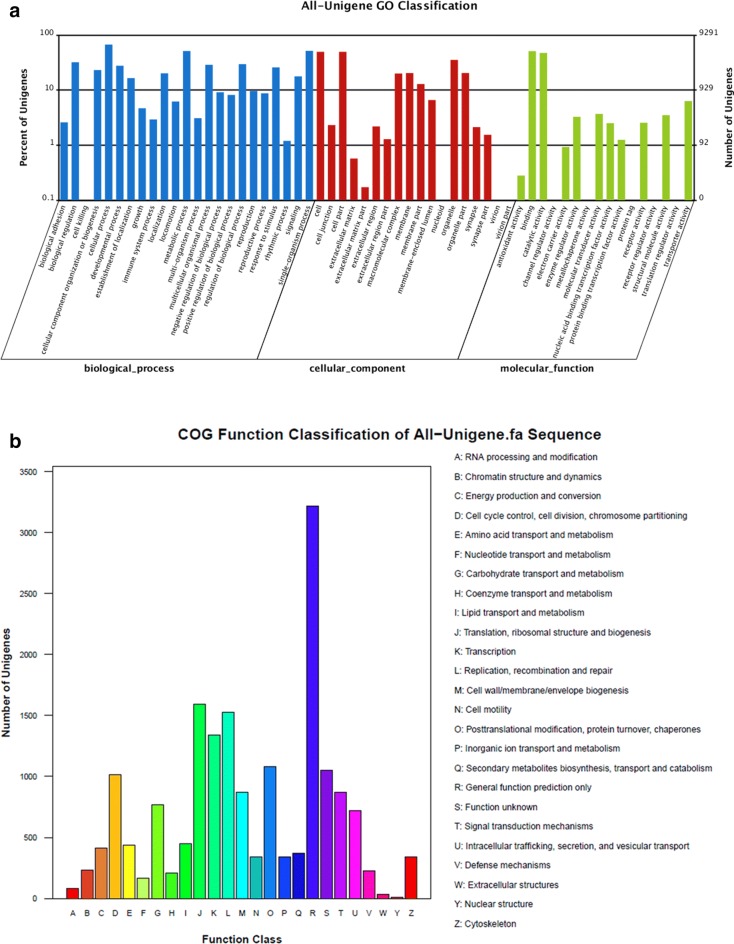
Functional classification of all unigenes from transcriptome sequencing results. **a** GO analysis of all unigenes in Sf9 cells. **b** COG function classification of all unigenes in Sf9 cells

A total of 9291 (34.81%) unigenes were classified into 3 main GO categories (biological process, cellular component, and molecular function) and 57 subcategories (Fig. 3a, Additional file 1: Table S1). The large part of unigenes were assigned to cellular process, with a percentage of 14.98%, then followed by single-organism process (11.51%) and metabolic process (11.44%). A total of 17,700 COG functional annotations were obtained and classified into 25 functional categories (Fig. 3b).

**Fig. 4:**
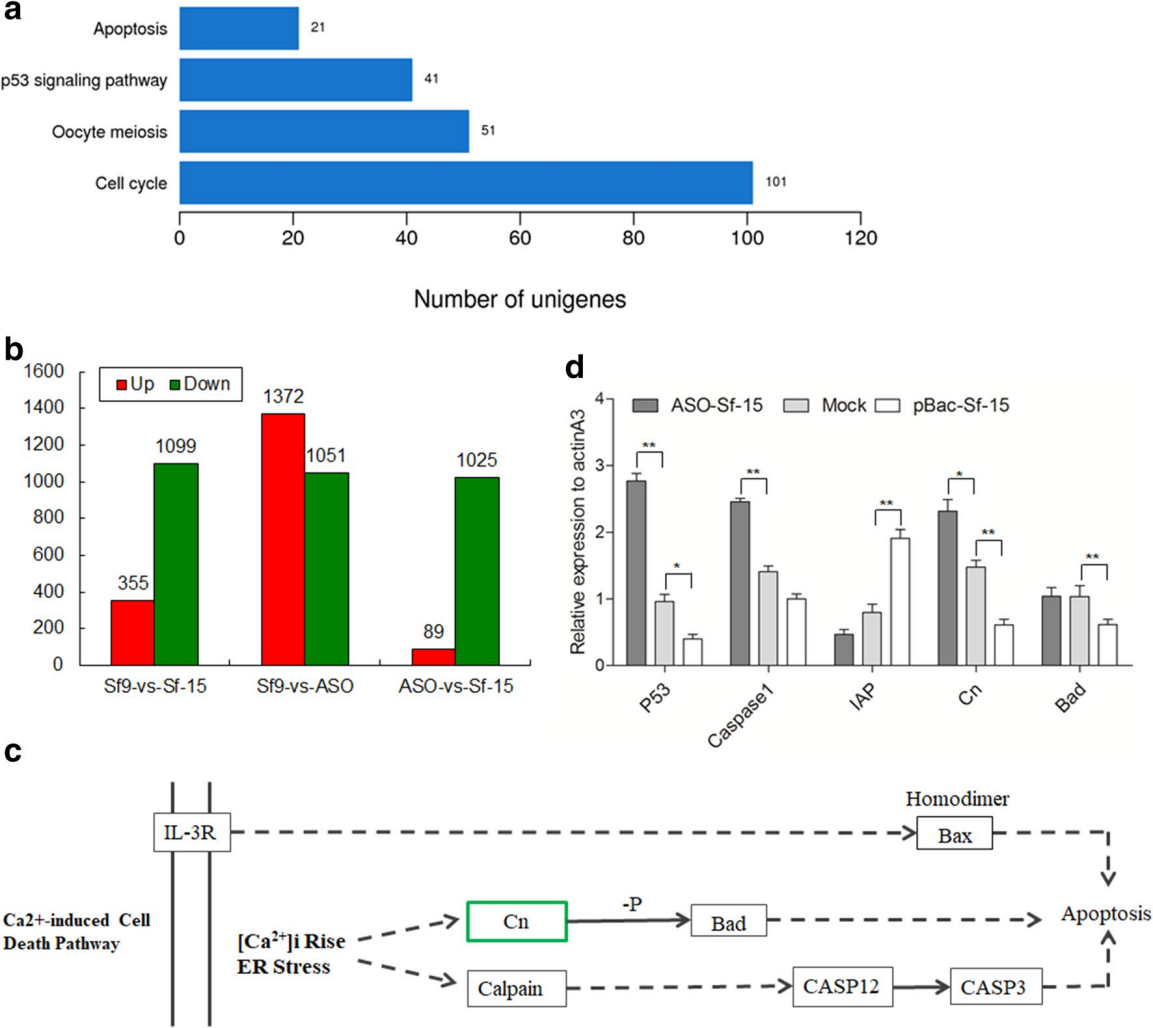
The potential targets of Sf-15. **a** KEGG pathway analysis of genes related to cell growth and death, which showed the numbers of genes in different signal pathways. **b** Numbers of genes with significantly varied expression upon Sf-15 repression and overexpression in Sf9 cells. Sf9 means normal Sf9 cells, Sf-15 represents cells transfected with the overexpression vector of *pBac[A3*-*EGFP*-*A3*-*BmSf*-*15]* (*pBac*-*Sf*-*15*). ASO means cells transfected with the antisense oligonucleotide of Sf-15. Red means the expression of genes were increased, green means the expression of genes were decreased. **c** Detailed information of genes in Ca^2+^ induced cell death pathway after overexpression of Sf-15 in Sf9 cells. Green box represented the expression of this gene was decreased upon Sf-15 overexpression but increased when Sf-15 was knocked down. **d** Quantitative real-time PCR results of genes in apoptosis pathway after transfection of Sf-15 antisense oligonucleotides (ASO) and overexpression vector *pBac[A3*-*EGFP*-*A3*-*Sf*-*15]* (*pBac*-*Sf*-*15*). *IAP* represented the inhibitor of apoptosis gene. *actinA3* was used as an internal control

Expression analysis showed that 355 unigenes were significantly upregulated and 1099 were significantly downregulated when Sf-15 was overexpressed. While 1372 were upregulated and 1051 were downregulated upon Sf-15 repression (Fig. 4b, Additional file 1: Table S1). Further analysis showed that the expression of 1111 unigenes had reverse expression pattern under Sf-15 overexpression and repression, the expressin of 909 genes were significantly increased when Sf-15 was knocked down, while 202 unigenes were significantly decreased when Bm-15 was repressed (Additional file 1: Table S1 and Additional file 2: Fig. S1). Interestingly, 21 genes of the apoptosis pathway were upregulated upon Sf-15 repression (Fig. 4a and Additional file 1: Table S1), but with reverse expression pattern when Sf-15 was overexpressed, which indicated Sf-15 might participate in the apoptosis pathway.

RT-PCR was carried out to further verify the sequencing results. We found that the expression of *p53* and *caspase*-*1* were increased when Sf-15 was knocked down, but decreased when Sf-15 was ectopic expressed in Sf9 cells. While the apoptosis inhibitor (*IAP*) gene had a contrary expression pattern (whose expression was decreased upon Sf-15 repression and increased when Sf-15 was overexpressed) (Fig. 4d), which indicated that Sf-15 might play roles in the process of cell growth and apoptosis.

### Sf-15 accelerate cell proliferation by targeting to *Cn* gene

To further study the targets of Sf-15, KEGG pathway of differentially expressed genes were analyzed, we found that 2 of the 21 apoptosis-related genes were significantly increased when Bm-15 was knocked down, especially the *Cn* gene (*PPP3C*, the catalytic subunit of *PP2B*, which encode a calcium-dependent serine/threonine phosphatase), with the log2 ratio of 7.5 (Fig. 4c). Quantitative real-time PCR results of *Cn* gene confirmed the results of transcriptome sequencing (Fig. 4d), which indicated that *Cn* might be a functional target of Sf-15. In the Ca^2+^ induced cell death pathway, *Bad* (Bcl-2/Bcl-xL-associated death promoter) was the downstream gene of *Cn*, the protein of which can be dephosphorylated by *Cn*. So the change of *Bad* was also detected by real-time PCR, results showed that the expression of *Bad* was decreased when Sf-15 was overexpressed, but with little variation upon Sf-15 repression, which was consistent with the transcriptome sequencing result, which indicated that *Cn* might regulate its downstream gene *Bad* not only in mRNA level but in the level of protein dephosphorylation. Further analysis showed that there is no sequence complement between Sf-15 and *Cn*, then how they interact with each other need much more evidence.

## Discussion

Regulation of apoptosis is essential for many developmental processes (Testa 2004). Recent years, besides protein coding genes, the non-coding RNAs were also found to be participated in cell growth and apoptosis in different organisms. For example, Gas5 (growth arrest specific 5), a tumor-suppressive long noncoding RNA, and a small nucleolar RNA (snoRNA) host gene similar to UHG (U22 host gene), encoded 10 human box C/D snoRNAs, can potently upregulates the transcription of tumor necrosis factor (TNF)-related apoptosis inducing ligand (*TRAIL*) by inducing H3K4 methylation/H3K27 demethylation. Overexpression of Gas5 transcripts were shown to induce growth arrest and apoptosis in several mammalian cell lines [12, 33, 39, 40]. Moreover, in *Drosophila*, the GAS5-origined scaRNA Dm46E3 had reverse expression pattern to its antisense protein coding gene *eiger* (who plays a key role in cell differentiation, apoptosis and immune response), might participate in embryogenesis of *Drosophila* through target to U1b snRNA, which indicated the novel role of snoRNAs in cell growth [41].

In our study, knocking down the C/D box snoRNA Sf-15 inhibited the proliferation and induced apoptosis and death of Sf9 cells. Then the expression of a serine/threonine-protein phosphatase 2B (*PP2B*) catalytic subunit gene *Cn*, was significantly increased upon Sf-15 depression. *Cn* is a Ca^2+^-dependent protein phosphatase (also known as *calcineurin*), which can catalyze the dephosphorylation of its downstream gene Bcl-2/Bcl-xL-associated death promoter (*Bad*), the dephosphorylated Bad can interact with Bcl-XL to initiate cell apoptosis and death [42]. The accumulation of *Cn* upon Sf-15 repression might increase the dephosphorylation level of Bad and initiate apoptosis and death of Sf9 cells. To investigate the mechanism that how Sf-15 interact with *Cn*, the sequences of Sf-15 and *Cn* were aligned in both strands, the secondary structure of them were also compared. But there was low potential of reverse complement between Sf-15 and *Cn* (data not shown). Moreover, we also analyzed whether Sf-15 had the potential to be precursor of miRNAs, but the answer was no. So how Sf-15 interact with *Cn*, and how Sf-15 participate in apoptosis through Ca^2+^-induced apoptosis pathway still need much more evidence.

Therefore, we hypothesize that Sf-15 might participate in the Ca^2+^ induced apoptosis pathway through regulating the expression of *Cn* and its downstream genes. Exploring how this snoRNA-mediated activity in apoptosis will be of great interest. Our finding of the apoptotosis-related snoRNA reveals a novel layer of snoRNA modulation in cell death program.

## Conclusions

More and more evidence showed that besides guiding 2′-*O*-methylation and pseudouridylation of rRNAs, snoRNAs can also interact with mRNAs, such as to be precursor of miRNAs, or compete with splicing factors at the splicing sites of pre-mRNAs. Here we found that knocking down of C/D box snoRNA Sf-15 induced apoptosis and death of Sf9 cells, further analysis showed that a Ca^2+^-induced cell death pathway gene *Cn* might be a potential target of Sf-15, which provide new clues for the functional excavation of snoRNAs in the future.

## Methods

### Cell culture and transfection

Sf9 cells were maintained in Grace medium (Thermo Fisher Scientific) containing 10% fetal bovine serum (FBS) (Thermo Fisher Scientific) at 28 °C. Cells were seed in 24-well plates to 70–80% confluency and further transfected with 2 μg of plasmid DNA or 50 nmol//L antisense oligonucleotides (ASO) employing Lipofectamine LTX and Plus Reagents (Thermo Fisher Scientific).

### RNA immunoprecipitation (RIP)

RNA immunoprecipitation (RIP) was followed the protocol of Selth et al. [43] with some modifications. Approximately 10^7^ Sf9 cells were cross-linked with 1% formaldehyde, the immunoprecipitation of Fibrillarin-containing protein complex was prepared with Protein G agarose (Roche) with the monoclonal antibody of Fibrillarin (Abcam). RNAs were isolated by phenol/chloroform/isoamylol. cDNA was generated by reverse transcription with 50 ng random hexamer primers from 2 μg total RNA using the Reverse Transcription System (Promega). PCR was performed as following, 94 °C for 5 min, then at 94 °C for 30 s, 55 °C for 30 s, and 72 °C for 30 s with 30 cycles. Primers used for Sf-15 was in the Additional file 2: Table S2. PCR products were detected by 2% agarose gel. RNA that being pulled down by serum was used as negative control, RNA that being extracted from cell lysate before immunoprecipitation (named as input) was used as the positive control.

### Nuclear and cytoplasmic RNA isolation and RT-PCR

The nuclear and cytoplasmic RNAs of Sf9 cells were extracted by Cytoplasmic & Nuclear RNA Purification Kit (Norgene), cDNAs were generated with 50 ng random hexamer primers (for ncRNAs) or oligo d(T)_15_ primers (for protein-coding genes) using the Reverse Transcription System (Promega). PCR was performed as following, 94 °C for 5 min, then at 94 °C for 30 s, 55 °C for 30 s, and 72 °C for 30 s with 25 cycles. The primers used for Sf-15, U6 and *actinA3* are listed in Additional file 2: Table S2. U6 and *actinA3* were used as nuclear and cytoplasmic control, respectively. PCR products were detected by 2% agarose gel.

### Fluorescent in situ hybridization (FISH)

FISH of Sf-15 in Sf9 cells was performed according to the method of Querido et al. [44]. Probe of Sf-15 used for in situ hybridization was as the following, CATCCAAGGAAGTTTGAAATGCGTGGTAATCATGGTTGCGCATTCATAAAGCATTGTA with Cy3-labeled at the 5′ end (TaKaRa). U3 was used as an internal control with FITC-labeled at the 5′ end (TaKaRa), nucleus was stained with DAPI. Localization of the Sf-15 was analyzed by fluorescent microscopy using laser scanning confocal microscope TE2000-E (Nikon).

### Plasmids construction and probe synthesis

Sf-15 was sub-cloned into the *pBac[A3*-*EGFP]* to construct the overexpression vector. Firstly, the *actinA3* promoter was cloned from the plasmid of *pBac[A3*-*EGFP]* with endonuclease BamHI and NocI, the fragment of Sf-15 was cloned from cDNA of Sf9 with NocI and KpnI at the 5′ and 3′ ends. *actinA3* promoter and Sf-15 were sub-cloned into the *pFBDM* vector, which constructed the vector of *pFBDM[A3*-*Sf*-*15]*. Next, *pFBDM[A3*-*Sf*-*15]* was digested by BamHI and BglII and ligated to *pBac[A3*-*EGFP]*, which constructed the *pBac[A3*-*EGFP*-*A3*-*Sf*-*15]* vector. These constructs were identified by sequencing performed by a commercial service provider (AuGCT Biotech).

To knock down the expression of Sf-15, the antisense oligonucleotides (ASO) of Sf-15 were used. ASO are designed in a 5-10-5 gapmer configuration (a chimeric antisense oligonucleotide that contains a central block of deoxynucleotide monomers sufficiently long to induce RNase H cleavage, gapmer is flanked by blocks of 2′-*O*-methoxyethol (MOE) modified ribonucleotides or other artificially modified ribonucleotide monomers can protect the internal block from nuclease degradation [45]), with the sequence of ugguuGCGCATTCATaaagc, the first and last five nucleotides were ribonucleic acids with MOE modification, the middle ten nucleotides were deoxyribonucleic acids, and the total probe had phosphorothioate (PS) chemical modification with Cy5-labeled at the 5′ end.

### Cell proliferation and apoptosis assays

Cell proliferation rate was detected using the Enhanced Cell Counting Kit-8 (Beyotime). Sf9 cells were transfected with *pBac[A3*-*EGFP*-*A3*-*Sf*-*15]* vector and ASO to overexpress and knock down of Sf-15, cells were collected at 6, 12, 24, 36, 48, 60, 72, 84, 96, 108, 120 and 126 h after transfection, 2000 cells were seeded in a 96-well plate in 100 μl of culture medium and incubated with 10 μl WST-8 (2-(2-methoxy-4-nitrophenyl)-3-(4-nitrophenyl)-5-(2,4-disulfophenyl)-2H-tetrazolium, monosodium salt) mixture, the analogue of MTT (3-(4,5-Dimethylthiazol-2-yl)-2,5-diphenyltetrazolium bromide), for 2 h. The absorbance of each sample was measured using a microplate reader at a wavelength of 450 nm. Each timepoint had three samples, each sample had three repeats.

Cell apoptosis detected by DAPI staining was carried out as following, Sf9 cells were collected after 72 h transfection of Sf-15 ASO, the nucleus was stained with DAPI, the chromatin condensation and apoptotic bodies were captured by fluorescent microscopy TE2000-E (Nikon).

The apoptosis rates of Sf9 cells were detected with the Annexin V-FITC cell apoptosis detection kit (Beyotime). 1 × 10^5^ cells were collected and stained with Annexin V-FITC and Propidium Iodide (PI), cells were incubated at room temperature for 20 min in dark place, then the apoptosis rates were detected on FC-500 flow cytometer (Beckman Coulter).

### Quantitative real-time PCR

Total RNA was extracted from different samples of Sf9 cells with TRIzol (Thermo Fisher Scientific) method. Synthesis of cDNA was performed with 2 μg of total RNA and 50 ng random hexamer primers (for ncRNAs) or oligo d(T)_15_ primers (for protein-coding genes) using the Reverse Transcription System (Promega). The primers used for ncRNAs and protein coding genes are listed in Additional file 2: Table S2. Quantitative real-time PCR was carried out using the FS Universal SYBR Green Master (Roche) on the CFX96™ Real-Time PCR Detection System (Bio-Rad). The thermal cycling consists a denaturation step at 95 °C for 10 min, then 40 cycles at 95 °C for 15 s, 55/58 °C for 30 s, and 72 °C for 30 s. Single PCR product was confirmed with the heat dissociation protocol at the end of the PCR cycles. The target gene abundance in each sample was normalized based on *U6* (for ncRNAs) or *actinA3* (for protein-coding genes) levels using the formula ΔCt = Ct target gene − Ct*U6* or *actinA3*. All experiments were conducted in three independent triplicates, each sample had three repeats.

### Illumina sequencing and de novo assembly

Total RNAs were extracted from normal Sf9 cell as well as cells being transfected with Sf-15 ASO and *pBac[A3*-*EGFP*-*A3*-*Sf*-*15]* with TRIzol method. Transcriptome sequencing was performed using Illumina HiSeq™ 2000. Raw reads with 3′-adaptors and repeating reads were removed, while nucleotides with a quality score lower than 20 were trimmed from the end of raw reads. Then, de novo assembly of the clean reads was conducted with Trinity (release-20130225), TGICLL (version 2.1) and Phrap (Release 23.0) programs to generate non-redundant unigenes.

### Bioinformatic analysis

Clean reads were aligned to NCBI non-redundant (NR) protein database, Swiss-Prot, KEGG, and Cluster of Orthologous Group (COG) databases using Blastx (E-value ≤ 1E−05). Unigene sequences were aligned to protein databases (NR, Swiss-Prot, KEGG and COG) to retrieve proteins with the highest sequence similarity to the given unigenes as their protein functional annotations. Furthermore, Blast2GO (version 2.5.0) [46] was used to obtain Gene Ontology (GO) annotation of unigenes with NR database. The WEGO software [47] was then used to perform functional classification of GO term for all unigenes and to comprehend the distribution of gene functions. The unigene sequences were aligned to the COG database to predict and classify possible functions. Pathway assignments were performed according to the KEGG database. The calculation of unigene expression was based on the FPKM method. We named genes with significantly differential expression when the log2 ratio of FPKM is more than 1. Genome sequence of *B*. *mori* and *S*. *frugiperda* were downloaded from SilkDB [48] and SPODOBASE [49, 50].

Sequence identity of Bm-15 between *B*. *mori* and *S*. *frugiperda* were aligned with the software of ClusterW (version 1.83). The secondary structure of Sf-15 was predicated by Mfold (Version 3.6) [51].

## Additional files

**Additional file 1: Table S1.** Differentially expressed gene list upon Sf-15 overexpression and repression. ASO and Sf-15 means cells were transfected with antisense oligonucleotides of Sf-15 and overexpression vector *pBac[A3-EGFP-A3-Sf-15]*, respectively.

**Additional file 2: Fig. S1.** Functional classification of genes with reverse expression pattern upon Bm-15 repression and overexpression. (A) GO classification of genes with expression being increased as Sf-15 was knocked down but decreased when Sf-15 was overexpressed. (B) GO classification of genes with expression being decreased when Sf-15 was knocked down but increased when Sf-15 was overexpressed. (C) Statistic analysis of the numbers of genes in figure A. (D) Statistic analysis of the numbers of genes in figure B. ASO and Sf-15 means cells were transfected with antisense oligonucleotides of Sf-15 and overexpression vector *pBac[A3-EGFP-A3-Sf-15]*, respectively. Up means the expression of genes were increased, down means the expression of genes were decreased. **Table S2.** Primer set used in the experiment. **Table S3.** Unigenes were subjected to Blastx against public protein database in Sf9 cells.

## Abbreviations

ncRNA: non-coding RNA
snoRNA: small nucleolar RNAs
rRNA: ribosomal RNA
snRNA: small nuclear RNAs
*Spodoptera frugiperda*: *S. frugiperda*
*B*. *mori*: *Bombyx mori*
Cn(PPP3C): serine/threonine protein phosphatase 3, catalytic subunit
5-HT_2C_R: serotonin receptor 2C
NSCLC: non-small cell lung cancer
FBS: fetal bovine serum
RIP: RNA immunoprecipitation
FISH: fluorescent in situ hybridization
WST: 2-(2-methoxy-4-nitrophenyl)-3-(4-nitrophenyl)-5-(2,4-disulfophenyl)-2H-tetrazolium, monosodium salt
MTT: 3-(4,5-dimethyl-2-thiazolyl)-2,5-diphenyl-2-H-tetrazolium bromide
DAPI: 4′,6-diamidino-2-phenylindole
ASO: antisense oligo nucleotide
NR: non-redundant
COG: Cluster of Orthologous Group
GO: Gene Ontology
IAP: apoptosis inhibitor
Bcl2: B-cell lymphoma 2
Apaf1: apoptotic protease-activating factor 1
Gas5: growth arrest specific 5
TNF: tumor necrosis factor
TRAIL: tumor necrosis factor-related apoptosis inducing ligand
