# Supplementary material for: Small nucleolar RNA Sf-15 regulates proliferation and apoptosis of Spodoptera frugiperda Sf9 cells
Source: BMC Mol Biol. 2019 Apr 11;20:12. doi: 10.1186/s12867-019-0128-9 (PMC6458620; doi:10.1186/s12867-019-0128-9)
Supplement: Supplementary file 2 — Additional file 2: Fig. S1. Functional classification of genes with reverse expression pattern upon Bm-15 repression and overexpression. (A) GO classification of genes with expression being increased as Sf-15 was knocked down but decreased when Sf-15 was overexpressed. (B) GO classification of genes with expression being decreased when Sf-15 was knocked down but increased when Sf-15 was overexpressed. (C) Statistic analysis of the numbers of genes in figure A. (D) Statistic analysis of the numbers of genes in figure B. ASO and Sf-15 means cells were transfected with antisense oligonucleotides of Sf-15 and overexpression vector pBac[A3-EGFP-A3-Sf-15], respectively. Up means the expression of genes were increased, down means the expression of genes were decreased. Table S2. Primer set used in the experiment. Table S3. Unigenes were subjected to Blastx against public protein database in Sf9 cells. [file 12867_2019_128_MOESM2_ESM.pdf]

Additional file 2:

Small nucleolar RNA Sf-15 regulates proliferation and apoptosis of *Spodoptera frugiperda* Sf9 cells

Bo Wu, Lei Huang, Wujie Qiu, Xiao Liu, Yawen Shen, Yiping Lu, Zonglin Yang, Xinmei Li, Bin Cui, Shidong Xu, Huili Qiao, Reng Qiu, Lunguang Yao, Yunchao Kan, Dandan Li

Content

1. **Additional file 2: Fig. S1.** Functional classification of genes with reverse expression pattern upon Bm-15 repression and overexpression.

2. **Additional file 2: Table S2.** Primer set used in the experiment.

3. **Additional file 2: Table S3.** Unigenes were subjected to Blastx against public protein database in Sf9 cells.

A

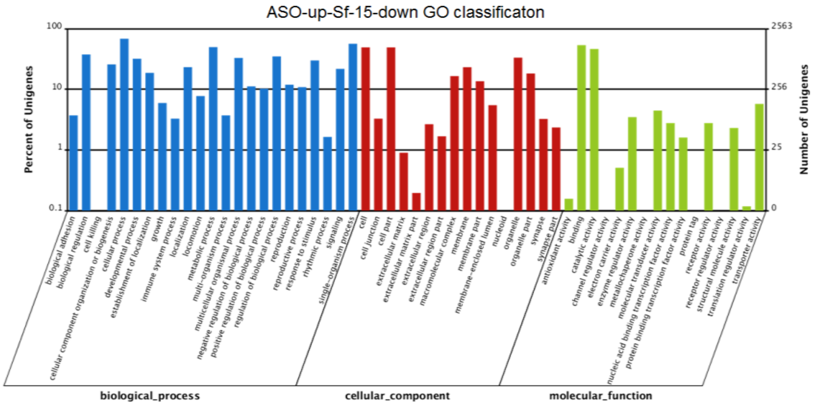

ASO-up-Sf-15-down GO classificaton
Percent of Unigenes
Number of Unigenes
100
10
1
0.1
2563
256
25
0
biological adhesion
biological regulation
cell killing
cellular component organization or biogenesis
cellular process
developmental process
establishment of localization
growth
immune system process
localization
locomotion
metabolic process
multi-organism process
multicellular organismal process
negative regulation of biological process
positive regulation of biological process
regulation of biological process
reproduction
reproductive process
response to stimulus
rhythmic process
signaling
single-organism process
cell
cell junction
cell part
extracellular matrix
extracellular matrix part
extracellular region
extracellular region part
macromolecular complex
membrane
membrane part
membrane-enclosed lumen
nucleoid
organelle
organelle part
synapse
synapse part
antioxidant activity
binding
catalytic activity
channel regulator activity
electron carrier activity
enzyme regulator activity
metallochaperone activity
molecular transducer activity
nucleic acid binding transcription factor activity
protein binding transcription factor activity
protein tag
receptor activity
receptor regulator activity
structural molecule activity
translation regulator activity
transporter activity
biological_process
cellular_component
molecular_function

B

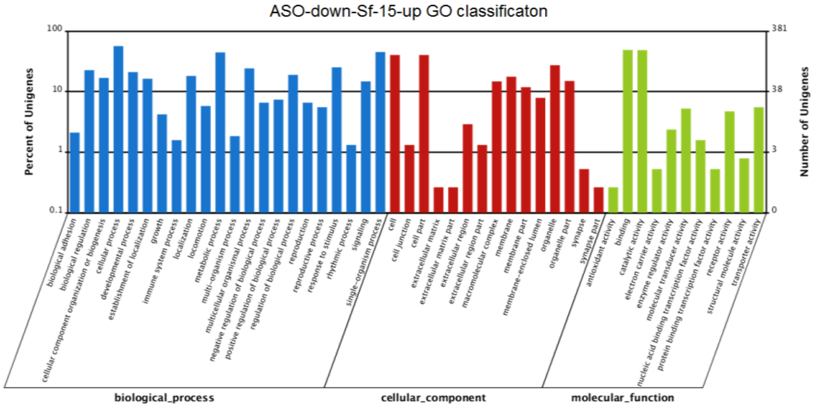

ASO-down-Sf-15-up GO classificaton
Percent of Unigenes
Number of Unigenes
100
10
1
0.1
381
38
3
0
biological adhesion
biological regulation
cellular component organization or biogenesis
cellular process
developmental process
establishment of localization
growth
immune system process
localization
locomotion
metabolic process
multi-organism process
multicellular organismal process
negative regulation of biological process
positive regulation of biological process
regulation of biological process
reproduction
reproductive process
response to stimulus
rhythmic process
signaling
single-organism process
cell
cell junction
cell part
extracellular matrix
extracellular matrix part
extracellular region
extracellular region part
macromolecular complex
membrane
membrane part
membrane-enclosed lumen
organelle
organelle part
synapse
synapse part
antioxidant activity
binding
catalytic activity
electron carrier activity
enzyme regulator activity
molecular transducer activity
nucleic acid binding transcription factor activity
protein binding transcription factor activity
receptor activity
structural molecule activity
transporter activity
biological_process
cellular_component
molecular_function

C

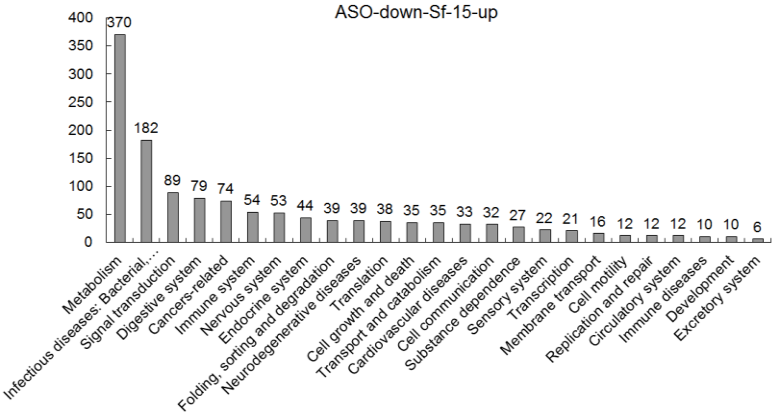

ASO-down-Sf-15-up
400
350
300
250
200
150
100
50
0
370
182
89
79
74
54
53
44
39
39
38
35
35
33
32
27
22
21
16
12
12
12
10
10
6
Metabolism
Infectious diseases: Bacterial,…
Signal transduction
Digestive system
Cancers-related
Immune system
Nervous system
Endocrine system
Folding, sorting and degradation
Neurodegenerative diseases
Translation
Cell growth and death
Transport and catabolism
Cardiovascular diseases
Cell communication
Substance dependence
Sensory system
Transcription
Membrane transport
Cell motility
Replication and repair
Circulatory system
Immune diseases
Development
Excretory system

D

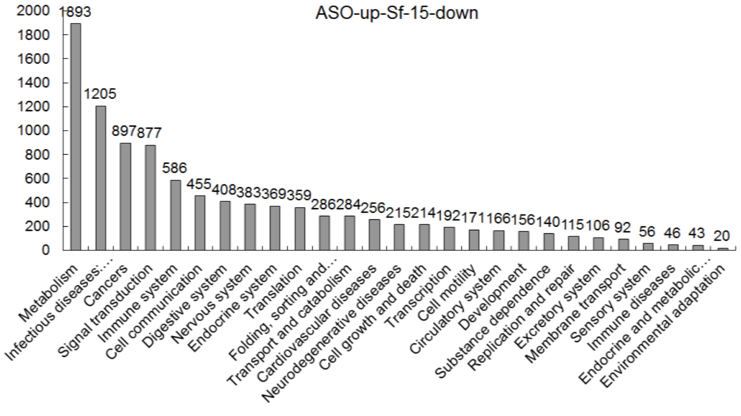

ASO-up-Sf-15-down
2000
1800
1600
1400
1200
1000
800
600
400
200
0
1893
1205
897
877
586
455
408
383
369
359
286
284
256
215
214
192
171
166
156
140
115
106
92
56
46
43
20
Metabolism
Infectious diseases:…
Cancers
Signal transduction
Immune system
Cell communication
Digestive system
Nervous system
Endocrine system
Translation
Folding, sorting and…
Transport and catabolism
Cardiovascular diseases
Neurodegenerative diseases
Cell growth and death
Transcription
Cell motility
Circulatory system
Development
Substance dependence
Replication and repair
Excretory system
Membrane transport
Sensory system
Immune diseases
Endocrine and metabolic…
Environmental adaptation

Additional file 2: Fig. S1. Functional classification of genes with reverse expression pattern upon Bm-15 repression and overexpression. (A) GO classification of genes with expression increased as Sf-15 was knocked down but decreased when Sf-15 was overexpressed. (B) GO classification of genes with expression repressed when Sf-15 was knocked down but induced when Sf-15 was overexpressed. (C) Statistic analysis of the numbers of genes in figure A. (D) Statistic analysis of the numbers of genes in figure B. ASO and Sf-15 means cells were transfected with antisense oligonucleotides of Sf-15 and overexpression vector *pBac[A3-EGFP-A3-Sf-15]*, respectively. Up means the expression of genes were increased, down means the expression of genes were decreased.

Additional file 2: Table S2. Primer set used in the experiment

**Gene**	**Forward primer**	**Reverse primer**
U6	CTAAAATTGGAACGATACAG	ATTTTGCGTGTCATCCTT
actinA3	TAGACAATGGCTCCGGTAT	CCAGTTAGTGACGATTCC
Sf-15	ATTCAATGATGATACAATGCTTTATG	AAATTCAGCCATCCAAGGAAGTT
Sf-15 primer for vector construction	CATGCCATGGATTCAATGATGATACAATG	GGGGTACCCCAAATTCAGCCATC
actinA3 promoter primer for vector construction	CGGGATCCCGCGTTACCATATATGGTGA	CATGCCATGGCCCGTACGAGTCCTTCT
P53	TATGATATGCCGTATGAAGAACTGG	ATTCGACAATTGATGCGGAT
Apoptosis inhibitor 5	CAAGAAACTTTTGGAGTTGCAG	CATGTTGTGTGCACTGGATAACT
Caspase 1	ATGCTGGACGGAAAACAAGACAA	GGCATTTCTATCAACTGGCATCCTA
Cn	TCACCCATACTGGTTGCC	CTGAGATTTGCTTCCTCC
Bad	CGTGAGAATGCGTAGAAA	ACGAGACCACCGTAACAA

Additional file 2: Table S3. Unigenes were subjected to Blastx against public protein database in Sf9 cells

Public protein database	NR	NT	Swiss-Prot	KEGG	COG	GO	ALL
Numbers	19,823	21,957	15,313	13,606	7,477	9,291	26,693
